# Overall survival according to immunotherapy and radiation treatment for metastatic non-small-cell lung cancer: a National Cancer Database analysis

**DOI:** 10.1186/s13014-019-1222-3

**Published:** 2019-01-28

**Authors:** Corey C. Foster, David J. Sher, Chad G. Rusthoven, Vivek Verma, Michael T. Spiotto, Ralph R. Weichselbaum, Matthew Koshy

**Affiliations:** 1 Department of Radiation and Cellular Oncology, The University of Chicago Medicine, 5758 S. Maryland Avenue, MC 9006, Chicago, IL 60637 USA; 2 Department of Radiation Oncology, UT Southwestern Medical Center, Harold C. Simmons Comprehensive Cancer Center, Radiation Oncology Building, 2280 Inwood Road, Dallas, TX 75390-9303 USA; 3 Department of Radiation Oncology at the Anschutz Medical Campus, University of Colorado School of Medicine, 1655 Aurora Court, Suite 1032, Aurora, CO 80045 USA; 4 Department of Radiation Oncology, Allegheny General Hospital, 320 E North Ave, Pittsburgh, PA 15212 USA; 5 Department of Radiation Oncology, University of Illinois at Chicago, Outpatient Care Center, 1801 West Taylor Street, Chicago, IL 60612 USA

**Keywords:** Non-small-cell lung cancer, National Cancer Database, Immunotherapy, Stereotactic radiotherapy, Radioimmunotherapy

## Abstract

**Background:**

Preclinical studies suggest enhanced anti-tumor activity with combined radioimmunotherapy. We hypothesized that radiation (RT) + immunotherapy would associate with improved overall survival (OS) compared to immunotherapy or chemotherapy alone for patients with newly diagnosed metastatic non-small-cell lung cancer (NSCLC).

**Methods:**

The National Cancer Database was queried for patients with stage IV NSCLC receiving chemotherapy or immunotherapy from 2013 to 2014. RT modality was classified as stereotactic radiotherapy (SRT) to intra- and/or extracranial sites or non-SRT external beam RT (EBRT). OS was analyzed using the Kaplan-Meier method and Cox proportional hazards models.

**Results:**

In total, 44,498 patients were included (13% immunotherapy, 46.8% EBRT, and 4.7% SRT). On multivariate analysis, immunotherapy (hazard ratio [HR]:0.81, 95% confidence interval [CI]:0.78–0.83) and SRT (HR:0.78, 95%CI:0.70–0.78) independently associated with improved OS; however, the interaction term for SRT + immunotherapy was insignificant (*p* = 0.89). For immunotherapy patients, the median OS for no RT, EBRT, and SRT was 14.5, 10.9, and 18.2 months, respectively (*p* < 0.0001), and EBRT (HR:1.37, 95%CI:1.29–1.46) and SRT (HR:0.78, 95%CI:0.66–0.93) associated with OS on multivariate analysis. In the SRT subset, median OS for immunotherapy and chemotherapy was 18.2 and 14.3 months, respectively (*p* = 0.004), with immunotherapy (HR:0.82, 95%CI:0.69–0.98) associating with OS on multivariate analysis. Furthermore, for patients receiving SRT, biologically effective dose (BED) > 60 Gy was independently associated with improved OS (HR:0.79, 95%CI:0.70–0.90, *p* < 0.0001) on multivariate analysis with a significant interaction between BED and systemic treatment (*p* = 0.008).

**Conclusions:**

Treatment with SRT associated with improved OS for patients with metastatic NSCLC irrespective of systemic treatment. The high survival for patients receiving SRT + immunotherapy strongly argues for evaluation in randomized trials.

## Background

In the United States, lung cancer is the most common cause of cancer-related mortality with 234,000 new cases and 154,000 deaths from this disease expected in 2018 [1]. Of these new cases, approximately 85% will be non-small-cell lung cancer (NSCLC) [2] which is most commonly diagnosed after distant metastasis has already occurred [3]. Unfortunately, the 5-year overall survival (OS) for patients with stage IV NSCLC from 2007 to 2013 was just 5.2% [3]. Therefore, it is not surprising that there has been significant interest in the application of new treatment modalities and therapeutic combinations to improve prognosis for this group of patients, including the use of immunotherapy [2] and consolidative radiotherapy (RT) for select oligometastatic cases [4].

Immune checkpoint blockade targeting the PD-1/PD-L1 axis was approved for metastatic NSCLC based on the groundbreaking CheckMate 017 and CheckMate 057 trials [5, 6]. Since that time, the indications for immune checkpoint blockade in NSCLC have expanded to include first-line therapy for patients with metastatic disease and tumor PD-L1 expression ≥50% [7]. Additionally, an OS benefit for all PD-L1 categories receiving pembrolizumab plus cytotoxic chemotherapy compared to cytotoxic chemotherapy alone has been observed in the setting of metastatic NSCLC without *EGFR* or *ALK* mutations [8, 9]. Moreover, patients with any history of predominantly intracranial (61%) RT receiving pembrolizumab for advanced NSCLC had improved OS in a secondary analysis of the phase I KEYNOTE-001 trial [10]. These data combined with promising preclinical observations [11, 12] and the theoretical advantages of combined radioimmunotherapy [13] have promoted enthusiasm for concurrent or sequential RT plus immunotherapy in advanced NSCLC. Therefore, we hypothesized that RT plus immunotherapy would associate with improved OS for patients with stage IV NSCLC in the National Cancer Database (NCDB) and further examined the influence of RT technique and dose on OS.

## Methods

### Data source

Data were procured from Health Insurance Portability and Accountability Act-compliant, de-identified participant user files extracted from the NCDB in an institutional-review board exempt study. The NCDB is a joint project of the Commission on Cancer (CoC) of the American College of Surgeons and the American Cancer Society. It was established in 1989 and includes demographic and oncologic outcomes data from over 1500 CoC-accredited cancer programs. Notably, the NCDB captures over 70% of incident cancer diagnoses nationally with more than 34 million historical records to date [14, 15]. While the data used in this study are derived from a de-identified NCDB file, the American College of Surgeons and the CoC have not verified and are not responsible for the analytic or statistical methodology employed, or the conclusions drawn from these data by the investigators.

### Study population

The NCDB was queried for patients with histologically proven stage IV NSCLC (American Joint Committee on Cancer staging manual, 7th Edition) who had received chemotherapy or immunotherapy as a first course of treatment at a CoC-accredited center from 2013 to 2014. This timeframe was chosen to ensure consistency in immunotherapy coding as some agents previously coded as chemotherapy were reclassified as immunotherapy in the NCDB effective January 1, 2013 [16]. Additionally, restricting the cohort to those receiving systemic therapy prior to the end of 2014 allowed for sufficient follow-up for correlation with OS.

In total, 44,498 patients met these inclusion criteria with *n* = 38,691 (86.9%) receiving chemotherapy and *n* = 5807 (13.1%) receiving immunotherapy. Patients were then further classified as receiving no RT, stereotactic radiotherapy (SRT), or non-SRT external beam radiotherapy (EBRT). RT modality was defined using NCDB codes whereby SRT included any patient specifically coded as receiving “stereotactic radiosurgery, NOS,” “linac radiosurgery,” or “Gamma Knife.” Thus, patients defined as receiving SRT necessarily included individuals receiving RT to intra- and/or extracranial sites. All other patients receiving RT and not labeled with at least 1 of these SRT-defining codes were considered to have received EBRT regardless of prescription dose or RT fractionation.

### Statistical considerations

Statistical analyses were performed with SPSS version 24 (IBM Inc., Armonk, NY) and JMP statistical software version 13.0 (SAS Institute, Cary, NC). The chi-square test was used for comparisons of patient-, tumor-, and treatment-related factors that were grouped categorically for analytic purposes. Variables that were specifically compared between chemotherapy and immunotherapy patient subsets included sex, age, modified Charlson-Deyo comorbidity index [17, 18], tumor histology, race (white vs. non-white), insurance status, treating facility type, and RT modality. Cumulative RT dose in Gray (Gy) and the number of RT fractions were used to calculate biologically effective dose (BED) using the formula BED = *n* · *d* · (1 + *d*/10) where *n* = number of fractions, *d* = dose per fraction, and 10 is the assumed alpha/beta ratio for NSCLC tumors. BED was compared between groups using univariate logistic regression.

Kaplan-Meier methods were used for univariate survival analyses while the log-rank test was used to make OS comparisons between and/or among groups based on treatment modality. Multivariate survival analyses with resultant hazard ratios (HRs) and 95% confidence intervals (CIs) were performed using Cox proportional hazards regression. Covariates for multivariate analyses were identified a priori and included factors known to influence OS for metastatic NSCLC as well as factors significantly differing between chemotherapy and immunotherapy groups. A test for interaction between SRT and immunotherapy was performed by comparing HRs for the SRT and no RT groups as a function of systemic therapy using a multiplicative interaction model. A similar test for interaction between BED and systemic therapy was performed for patients receiving SRT with available dose/fractionation data. All statistical tests used two-sided hypothesis testing with a type I error < 0.05 defining statistical significance.

## Results

### Population characteristics

Patient-, tumor-, and treatment-related characteristics for the entire cohort and for the immunotherapy vs. chemotherapy subpopulations are displayed in Table 1. A total of 44,498 patients with stage IV NSCLC met inclusion criteria with *n* = 5807 (13.1%) receiving immunotherapy and *n* = 38,691 (86.9%) receiving chemotherapy. Compared to the chemotherapy group, the immunotherapy group contained a larger proportion of female patients, younger patients, and patients with adenocarcinoma histology. There was no significant difference in the proportion of patients receiving treatment at academic or non-academic centers as a function of systemic therapy (*p* = 0.48).

**Table 1:** Patient-, tumor-, and treatment-related characteristics

		All Patients (*n* = 44,498)		Immunotherapy (*n* = 5807)		Chemotherapy (*n* = 38,691)		*p*-value
		No.	%	No.	%	No.	%	
Sex	Male	24,693	55.5	3031	52.2	21,662	56.0	< 0.001
	Female	19,805	44.5	2776	47.8	17,029	44.0	
Age	18–59	13,849	31.1	1947	33.5	11,902	30.8	< 0.001
	60–69	15,487	34.8	2060	35.5	13,427	34.7	
	70–79	12,224	27.5	1499	25.8	10,725	27.7	
	80+	2938	6.6	301	5.2	2637	6.8	
Comorbidity	0	28,932	65.0	3889	67.0	25,043	64.7	< 0.001
	1	11,280	25.3	1456	25.1	9824	25.4	
	> 2	4286	9.6	462	8.0	3824	9.9	
Histology	Adenocarcinoma	26,202	58.9	4596	79.1	21,606	55.8	< 0.001
	Non-adenocarcinoma	18,296	41.1	1211	20.9	17,085	44.2	
Race	White	37,293	83.8	4950	85.2	32,343	83.6	< 0.001
	Non-white	7205	16.2	857	14.8	6348	16.4	
Insurance	Uninsured	1871	4.2	186	3.2	1685	4.4	< 0.001
	Insured	42,627	95.8	5621	96.8	37,006	95.6	
Facility Type	Academic	14,480	32.5	1913	32.9	12,567	32.5	0.48
	Nonacademic	30,018	67.5	3894	67.1	26,124	67.5	
Radiation	No Radiation	21,593	48.5	3344	57.6	18,249	47.2	< 0.001
	EBRT	20,821	46.8	2235	38.5	18,586	48.0	
	SRT	2084	4.7	228	3.9	1856	4.8	
Systemic Agent	Multi-agent chemotherapy	38,691	86.9					
	Immunotherapy	5807	13.1					

Regarding RT, *n* = 21,593 (48.5%) did not receive RT, *n* = 20,821 (46.8%) received EBRT (34.6% intracranial), and *n* = 2048 (4.7%) received SRT (81.3% intracranial). Of these, *n* = 20,031 (87.6%) had evaluable RT dose/fractionation information for BED calculations. The median dose for patients receiving EBRT was 30 Gy in a median of 10 fractions (interquartile range [IQR]: 10–15) while the median dose for patients receiving SRT was 22 Gy in a median of a single fraction (IQR: 1–4). The median BED for the SRT group was 60 Gy (IQR: 43.2–70.4) and was significantly higher than the median BED of 39 Gy (IQR: 39.0–50.7) for the EBRT group (*p* < 0.0001). Of patients in the SRT group with known dose/fractionation information, 34.5% received a BED > 60 Gy with this subset prescribed a median RT dose of 24 Gy in a median of a single fraction. The most common RT dose/fractionation prescriptions for patients receiving SRT to a BED ≤60 Gy vs. > 60 Gy are displayed in Table 2.

**Table 2:** Most frequent radiation prescriptions for patients receiving SRT with available dose/fractionation data (*n* = 1739)

BED ≤60 Gy (*n* = 1139)			BED > 60 Gy (*n* = 600)		
Dose/fractionation	BED	No. (%)	Dose/fractionation	BED	No. (%)
20 Gy in 1 fraction	60.0	257 (22.5)	24 Gy in 1 fraction	81.6	140 (23.3)
18 Gy in 1 fraction	50.4	203 (17.8)	21 Gy in 1 fraction	65.1	93 (15.5)
24 Gy in 3 fractions	43.2	89 (7.8)	22 Gy in 1 fraction	70.4	64 (10.7)
30 Gy in 5 fractions	48.0	77 (6.8)	50 Gy in 5 fractions	100.0	36 (6.0)

*Abbreviations*: *SRT* stereotactic radiotherapy, *BED* biologically equivalent dose, *Gy* Gray

### Survival

**Fig. 1:**
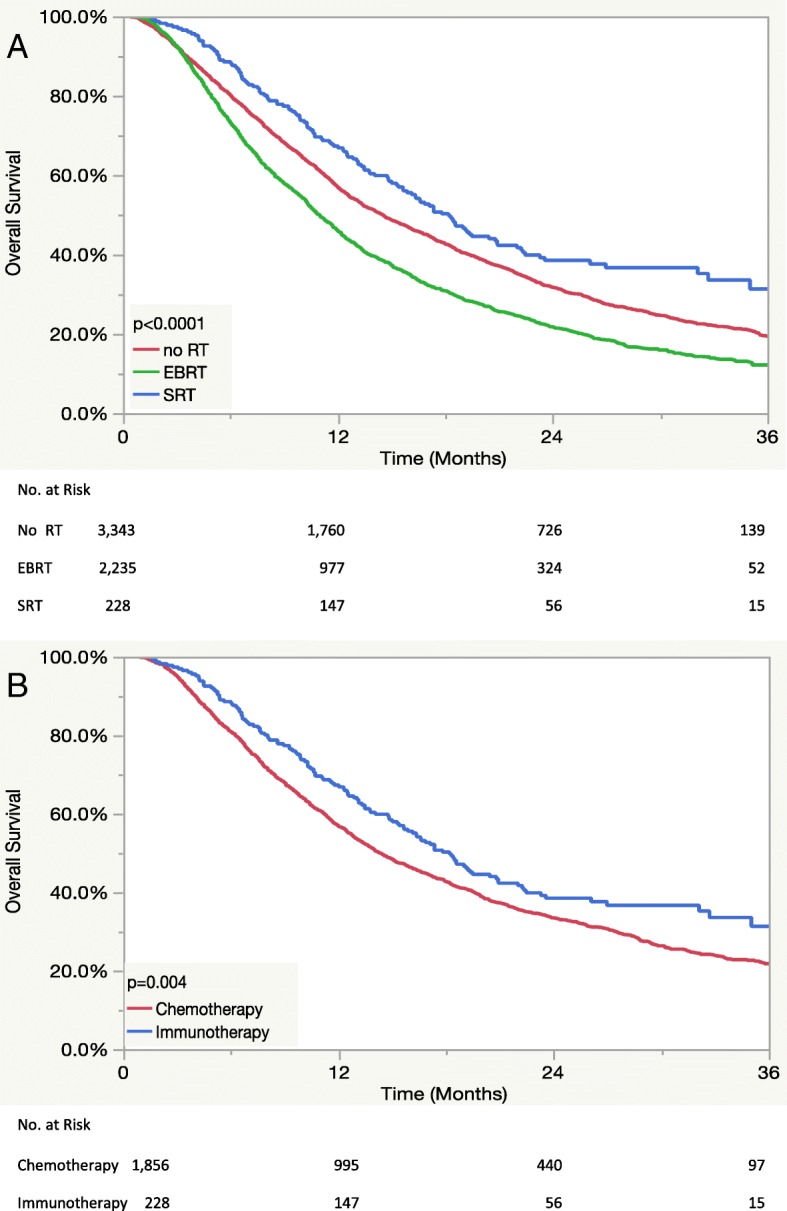
Kaplan-Meier curves displaying overall survival (**a**) as a function of radiation modality for patients receiving immunotherapy and (**b**) as a function of systemic therapy for patients receiving stereotactic radiotherapy. *Abbreviations:* RT = radiotherapy, EBRT = external beam radiotherapy, SRT = stereotactic radiotherapy

**Fig. 2:**
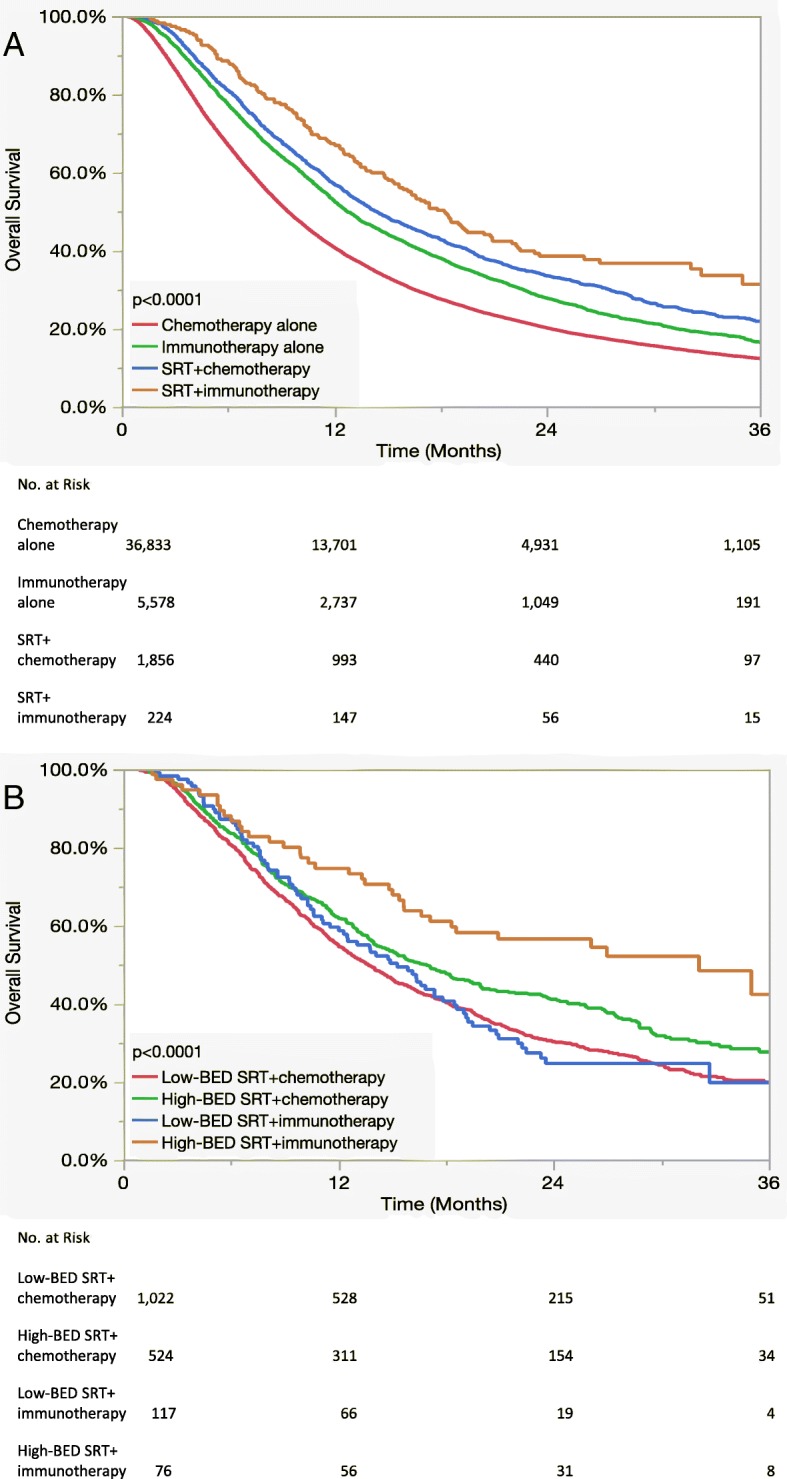
Kaplan-Meier curves displaying overall survival (**a**) as a function of combined radiation and systemic therapy and (**b**) as a function of biologically effective dose and systemic therapy for patients receiving stereotactic radiotherapy

**Fig. 3:**
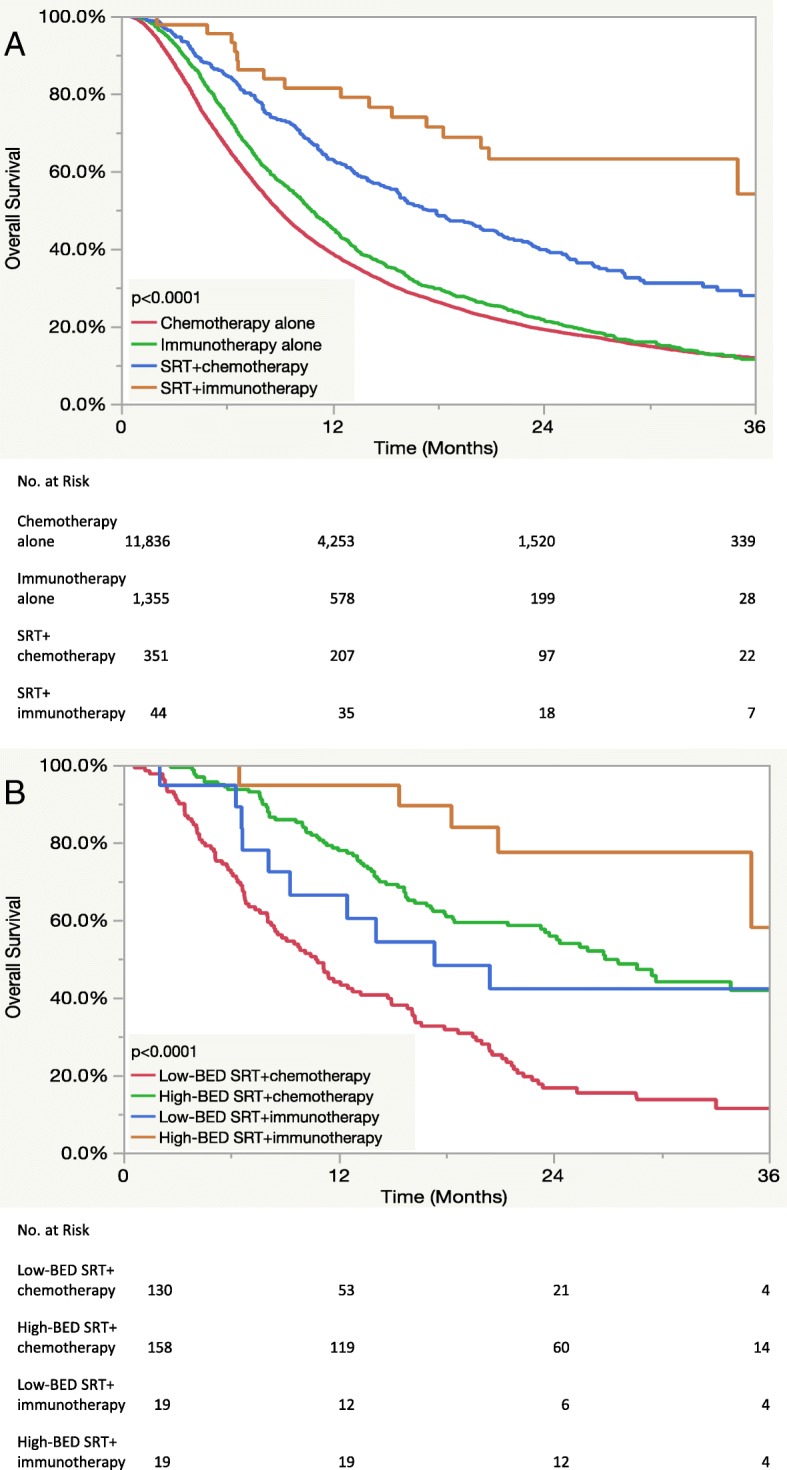
Kaplan-Meier curves displaying overall survival (**a**) as a function of combined radiation and systemic therapy and (**b**) as a function of biologically effective dose and systemic therapy for patients receiving extracranial stereotactic radiotherapy. *Abbreviations:* SRT = stereotactic radiotherapy, BED = biologically effective dose

The median survival of patients receiving immunotherapy was 17.3 months vs. 14.4 months in the chemotherapy group (*p* = 0.03). Kaplan-Meier plots investigating the relationship between combined systemic and RT modalities for the immunotherapy (*n* = 5807) and SRT (*n* = 2084) subpopulations are displayed in panels A and B of Fig. 1, respectively. Within the immunotherapy cohort, median OS was 14.5, 10.9, and 18.2 months (*p* < 0.0001) for patients receiving no RT, EBRT, and SRT, respectively. Meanwhile, within the SRT cohort, median OS was 18.2 months and 14.3 months (*p* = 0.004) for patients treated systemically with immunotherapy or chemotherapy, respectively. A Kaplan-Meier plot displaying OS as a function of systemic therapy with or without SRT is also displayed in panel A of Fig. 2 with this stratification significantly associating with OS (*p* < 0.0001). A similar plot displaying OS as a function of systemic therapy with or without extracranial SRT is displayed in panel A of Fig. 3 and also found a significant association with OS (*p* < 0.0001) with patients receiving extracranial SRT and immunotherapy having the best prognosis.

The results of a multivariate analysis for OS for the entire cohort are displayed in Table 3. The independent predictors with the largest impact on OS included Charlson-Deyo comorbidity score (HR:1.23, 95% CI:1.19–1.28 for scores ≥2 vs. 0, *p* < 0.0001), systemic therapy (HR: 0.81, 95% CI: 0.78–0.84 for immunotherapy vs. chemotherapy, *p* < 0.0001), and RT modality (HR: 0.78, 95% CI: 0.69–0.78 for SRT vs. no RT; HR: 1.16, 95% CI: 1.13–1.18 for EBRT vs. no RT, *p* < 0.0001). This same multivariate analysis performed while adjusting for intra- vs. extracranial RT continued to identify systemic therapy (HR: 0.87, 95% CI: 0.83–0.91 for immunotherapy vs. chemotherapy, p < 0.0001) and RT modality (HR: 0.60, 95% CI: 0.57–0.63 for SRT vs. EBRT, p < 0.0001) as independently associated with OS. Since RT site was also significantly associated with OS (HR: 1.12, 95% CI: 1.08–1.15 for intracranial vs. extracranial RT, *p* < 0.0001), results of the multivariate analysis were separately reported for the intra- and extracranial RT subgroups as displayed in Table 4. RT modality as well as systemic therapy continued to be independently associated with OS for both the intracranial and extracranial subgroups. Moreover, the association of combined immunotherapy and SRT with improved OS was persistent on multivariate analyses for the immunotherapy and SRT subgroups as respectively displayed in Tables 5 and 6. Specifically, for patients receiving immunotherapy, EBRT (HR: 1.37, 95% CI: 1.29–1.46) was associated with significantly reduced OS while SRT (HR: 0.78, 95% CI: 0.66–0.93) was associated with significantly improved OS (*p* < 0.0001 comparing no RT, EBRT, and SRT). Furthermore, for patients undergoing SRT, immunotherapy (HR: 0.82, 95% CI: 0.69–0.98, *p* = 0.031) associated with higher OS compared to chemotherapy. Despite the prolonged OS for patients receiving combined immunotherapy and SRT compared to patients receiving alternative combinations of systemic and RT modalities, the interaction term for SRT and immunotherapy did not reach significance (*p* = 0.89).

**Table 3:** Multivariate analysis including all patients (*n* = 44,498)

		Hazard Ratio	95% Confidence Interval	*p*-value
Sex	Male	1.00	Ref	< 0.0001
	Female	0.82	0.80–0.84	
Age	18–59	1.00	Ref	< 0.0001
	60–69	1.02	.099–1.05	
	70–79	1.05	1.02–1.08	
	80+	1.15	1.10–1.20	
Comorbidity	0	1.00	Ref	< 0.0001
	1	1.08	1.05–1.10	
	> 2	1.23	1.19–1.28	
Histology	Adenocarcinoma	1.00	Ref	< 0.0001
	Non-adenocarcinoma	1.14	1.12–1.16	
Race	White	1.00	Ref	< 0.0001
	Non-white	0.88	0.85–0.91	
Insurance	Uninsured	1.00	Ref	0.012
	Insured	0.92	0.88–0.98	
Facility Type	Academic	1.00	Ref	< 0.0001
	Nonacademic	1.16	1.13–1.18	
Systemic Therapy	Chemotherapy	1.00	Ref	< 0.0001
	Immunotherapy	0.81	0.78–0.84	
Radiation	No Radiation	1.00	Ref	< 0.0001
	EBRT	1.16	1.13–1.18	
	SRT	0.73	0.69–0.78

**Table 4:** Multivariate analysis as a function of radiated site

		Extracranial Radiation (*n* = 13,586)			Intracranial Radiation (*n* = 8624)		
		Hazard Ratio	95% Confidence Interval	*p*-value	Hazard Ratio	95% Confidence Interval	*p*-value
Sex	Male	1.00	Ref	< 0.0001	1.00	Ref	< 0.0001
	Female	0.85	0.81–0.89		0.82	0.78–0.86	
Age	18–59	1.00	Ref	0.087	1.00	Ref	< 0.0001
	60–69	0.98	0.94–1.03		1.10	1.04–1.16	
	70–79	1.00	0.95–1.05		1.27	1.19–1.35	
	80+	1.10	1.01–1.20		1.43	1.24–1.64	
Comorbidity	0	1.00	Ref	0.002	1.00	Ref	< 0.0001
	1	1.04	0.99–1.08		1.09	1.03–1.15	
	> 2	1.12	1.05–1.20		1.25	1.15–1.36	
Histology	Adenocarcinoma	1.00	Ref	< 0.0001	1.00	Ref	< 0.0001
	Non-adenocarcinoma	1.09	1.05–1.14		1.18	1.13–1.25	
Race	White	1.00	Ref	< 0.0001	1.00	Ref	< 0.0001
	Non-white	0.88	0.83–0.92		0.86	0.80–0.92	
Insurance	Uninsured	1.00	Ref	0.753	1.00	Ref	0.533
	Insured	1.02	0.92–1.12		1.04	0.93–1.16	
Facility Type	Academic	1.00	Ref	< 0.0001	1.00	Ref	< 0.0001
	Nonacademic	1.11	1.07–1.16		1.21	1.15–1.27	
Systemic Therapy	Chemotherapy	1.00	Ref	0.002	1.00	Ref	< 0.0001
	Immunotherapy	0.90	0.85–0.96		0.83	0.77–0.90	
Radiation	EBRT	1.00	Ref	< 0.0001	1.00	Ref	< 0.0001
	SRT	0.51	0.45–0.58		0.62	0.58–0.66

**Table 5:** Multivariate analysis for patients receiving immunotherapy (*n* = 5807)

		Hazard Ratio	95% Confidence Interval	*p*-value
Sex	Male	1.00	Ref	< 0.0001
	Female	0.79	0.75–0.84	
Age	18–59	1.00	Ref	< 0.0001
	60–69	1.08	1.01–1.17	
	70–79	1.14	1.05–1.24	
	80+	1.26	1.09–1.46	
Comorbidity	0	1.00	Ref	< 0.0001
	1	1.15	1.07–1.23	
	> 2	1.25	1.12–1.40	
Histology	Adenocarcinoma	1.00	Ref	< 0.0001
	Non-adenocarcinoma	1.15	1.06–1.23	
Race	White	1.00	Ref	0.012
	Non-white	0.89	0.82–0.98	
Insurance	Uninsured	1.00	Ref	0.079
	insured	0.86	0.72–1.02	
Facility Type	Academic	1.00	Ref	< 0.0001
	Nonacademic	1.26	1.18–1.34	
Radiation	No Radiation	1.00	Ref	< 0.0001
	EBRT	1.37	1.29–1.46	
	SRT	0.78	0.66–0.93

**Table 6:** Multivariate analysis for patients receiving SRT (*n* = 2048)

		Hazard Ratio	95% Confidence Interval	*p*-value
Sex	Male	1.00	Ref	0.001
	Female	0.83	0.75–0.92	
Age	18–59	1.00	Ref	0.058
	60–69	1.07	0.95–1.22	
	70–79	1.2	1.04–1.38	
	80+	1.24	0.96–1.58	
Comorbidity	0	1.00	Ref	0.062
	1	0.99	0.87–1.13	
	> 2	1.25	1.03–1.5	
Histology	Adenocarcinoma	1.00	Ref	< 0.001
	Non-adenocarcinoma	1.31	1.17–1.46	
Race	White	1.00	Ref	0.538
	Non-white	1.05	0.91–1.21	
Insurance	Uninsured	1.00	Ref	0.582
	insured	0.92	0.69–1.24	
Facility Type	Academic	1.00	Ref	0.088
	Nonacademic	1.1	0.99–1.22	
Systemic Therapy	Chemotherapy	1.00	Ref	0.031
	Immunotherapy	0.82	0.69–0.98	

**Fig. 4:**
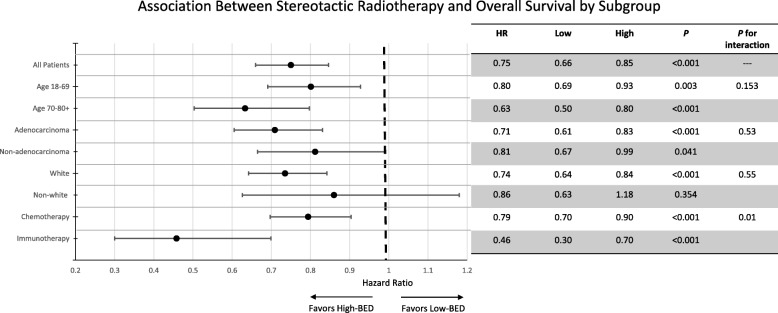
Forest plot for the impact of the biologically effective dose of stereotactic radiotherapy on overall survival by subgroup. *Abbreviations:* HR = hazard ratio, BED = biologically effective dose

The influence of BED (“low BED” ≤60 Gy vs. “high BED” > 60 Gy) [19] and systemic therapy on OS for patients receiving SRT with available dose/fractionation information (*n* = 1739) is displayed in panel B of Fig. 2. Notably, patients receiving high-BED SRT with immunotherapy had a median OS of 32.1 months compared to 16.6 months for high-BED SRT with chemotherapy, 15.3 months for low-BED SRT with immunotherapy, and 13.7 months for low-BED SRT with chemotherapy (*p* < 0.0001 when comparing all groups). A similar Kaplan-Meier plot showing OS using the same stratification for patients receiving specifically extracranial SRT is displayed in panel B of Fig. 3 and once again demonstrated the highest OS for patients receiving high-BED SRT and immunotherapy (*p* < 0.0001). On multivariate analysis including all SRT patients with available dose/fractionation data as displayed in Table 7, BED had the largest impact on OS of all covariates (HR:0.79, 95% CI:0.70–0.90, *p* < 0.0001), and systemic therapy was no longer significantly associated with OS after including BED in the regression (*p* = 0.51). Moreover, a significant interaction was observed between BED and systemic therapy (*p* = 0.008) as shown in the forest plot displayed in Fig. 4. Multivariate analysis results are separately reported for patients receiving extra- vs. intracranial RT in Table 8. This identified BED (HR: 0.34, 95% CI: 0.25–0.46, for high- vs. low-BED SRT, *p* < 0.0001) and systemic therapy (HR: 0.49, 95% CI: 0.28–0.82 for immunotherapy vs. chemotherapy, *p* = 0.01) as independently associated with OS for patients receiving extracranial RT.

**Table 7:** Multivariate analysis including patients who received stereotactic radiotherapy with dose/fractionation data (*n* = 1739)

		Hazard Ratio	95% Confidence Interval	*p*-value
Sex	Male	1.00	Ref	0.001
	Female	0.82	0.73–0.92	
Age	18–59	1.00	Ref	0.01
	60–69	1.12	0.97–1.29	
	70–79	1.27	1.09–1.48	
	80+	1.35	1.04–1.76	
Comorbidity	0	1.00	Ref	0.004
	1	1.02	0.89–1.18	
	> 2	1.41	1.15–1.73	
Histology	Adenocarcinoma	1.00	Ref	< 0.0001
	Non-adenocarcinoma	1.29	1.14–1.45	
Race	White	1.00	Ref	0.52
	Non-white	1.05	0.90–1.23	
Insurance	Uninsured	1.00	Ref	0.77
	Insured	0.95	0.69–1.32	
Facility Type	Academic	1.00	Ref	0.17
	Nonacademic	1.09	0.97–1.22	
Systemic Therapy	Chemotherapy	1.00	Ref	0.51
	Immunotherapy	1.08	0.86–1.37	
Biologically Effective Dose	≤60 Gy	1.00	Ref	< 0.0001
	> 60 Gy	0.79	0.70–0.90

**Table 8:** Multivariate analysis for patients with dose/fractionation data as a function of radiated site

		Extracranial Radiation (*n* = 326)			Intracranial Radiation (*n* = 1395)		
		Hazard Ratio	95% Confidence Interval	*p*-value	Hazard Ratio	95% Confidence Interval	*p*-value
Sex	Male	1.00	Ref	0.288	1.00	Ref	0.0006
	Female	0.85	0.64–1.14		0.80	0.70–0.91	
Age	18–59	1.00	Ref	0.104	1.00	Ref	0.042
	60–69	1.41	0.98–2.06		1.06	0.91–1.24	
	70–79	1.47	0.98–2.21		1.25	1.05–1.48	
	80+	2.09	0.97–4.10		1.30	0.96–1.71	
Comorbidity	0	1.00	Ref	0.481	1.00	Ref	0.009
	1	1.18	0.83–1.64		0.99	0.85–1.16	
	> 2	1.27	0.74–2.04		1.42	1.12–1.77	
Histology	Adenocarcinoma	1.00	Ref	0.376	1.00	Ref	< 0.0001
	Non-adenocarcinoma	1.14	0.85–1.53		1.35	1.18–1.55	
Race	White	1.00	Ref	0.561	1.00	Ref	0.750
	Non-white	1.13	0.74–1.66		1.03	0.86–1.22	
Insurance	Uninsured	1.00	Ref	0.82	1.00	Ref	0.697
	Insured	0.91	0.42–2.37		0.89	0.79–1.01	
Facility Type	Academic	1.00	Ref	0.871	1.00	Ref	0.084
	Nonacademic	1.03	0.76–1.40		1.12	0.99–1.27	
Systemic Therapy	Chemotherapy	1.00	Ref	0.01	1.00	Ref	0.886
	Immunotherapy	0.49	0.28–0.82		0.98	0.79–1.21	
Biologically Effective Dose	≤60 Gy	1.00	Ref	< 0.0001	1.00	Ref	0.538
	> 60 Gy	0.34	0.25–0.46		0.96	0.83–1.10	

## Discussion

Within the NCDB, patients with stage IV NSCLC receiving immunotherapy experienced improved OS compared to patients receiving chemotherapy. These findings are the first to demonstrate population-wide benefits with the use of immunotherapy in this setting. The median survival of 17.3 months and HR of 0.8 associated with immunotherapy in this NCDB cohort compares favorably to the HR of 0.81 and 16.7-month median survival in the PD-L1 ≥ 1% cohort receiving immunotherapy on the ongoing and recently presented phase III KEYNOTE-042 trial [20]. This similarity in survival outcomes adds validity to these results and demonstrates the efficacy of immunotherapy in patients treated outside of a randomized phase III study.

On subset analysis, patients undergoing predominantly intracranial SRT had improved OS compared to those who did not regardless of systemic therapy choice. Interestingly, among patients receiving immunotherapy, SRT associated with improved OS while non-SRT EBRT associated with reduced OS. BED significantly correlated with OS for patients undergoing SRT with individuals receiving a BED > 60 Gy in combination with immunotherapy having an impressive median OS of 32.1 months compared to just 13.7 months for individuals treated with low-BED SRT and chemotherapy. Although a synergistic interaction between SRT and immunotherapy was not identified among all patients receiving immunotherapy, a synergistic interaction between high BED and systemic therapy was identified among patients receiving SRT.

These data add to a growing body of literature attempting to clarify the potential role of SRT in the setting of metastatic NSCLC [4, 21, 22]. Specifically, our finding that patients receiving SRT experienced improved OS should be considered alongside the previously reported high progression-free survival (PFS) of 14.7 months and median OS of 20.4 months in a single-arm, phase II protocol of SRT with concurrent erlotinib for patients with stage IV NSCLC involving ≤6 sites after progression through first- or subsequent-line chemotherapy [22]. Moreover, compared to an expected median OS of just 11 months for stage IV NSCLC treated with a first-line platinum doublet [23], the median OS of 14.3 months for SRT + chemotherapy and 18.2 months for SRT + immunotherapy as observed in our report are quite promising. Although analyses investigating differences in OS based on the existence of oligometastatic versus polymetastatic disease are not possible using the NCDB, Gomez et al. have previously reported a high median PFS of 11.9 months after local consolidative therapy including SRT for patients with oligometastatic NSCLC involving ≤3 sites that had not progressed after first-line chemotherapy [4]. This again suggests that select patients with metastatic NSCLC may be appropriate candidates for an aggressive treatment paradigm incorporating local ablative therapy.

Preclinical studies have suggested enhanced anti-tumor activity for combined radioimmunotherapy including intracranial RT in mouse glioblastoma models [11, 12]. This observation in the laboratory was bolstered by a secondary analysis of the KEYNOTE-001 phase I trial wherein patients with advanced NSCLC receiving any prior RT (61% intracranial) followed by pembrolizumab had a significantly higher median OS of 10.7 months vs. 5.3 months for those receiving pembrolizumab without previous RT [10]. These data are encouraging when viewed as a foundation to support the use of RT as a strategy to induce response to immune checkpoint blockade for patients with metastatic NSCLC as the unselected overall response rate with single agent PD-1/PD-L1 blockade is only 17–19% [24, 25]. This possibility is further suggested by the results of a randomized phase II study of SRT and sequential pembrolizumab vs. pembrolizumab alone for patients with advanced NSCLC with the experimental arm having an overall response rate of 41% and a median PFS of 6.4 months compared to 19% and 1.8 months, respectively, for the control arm [26]. Our observed significantly prolonged median OS of 18.2 months for combined SRT and immunotherapy in the NCDB is consistent with these previously published data; however, evidence of a synergistic interaction between SRT and immunotherapy did not reach significance.

RT dose and fractionation may influence the complex relationship between RT-induced immunomodulation and anti-tumor response [13, 19]. Standard RT fractionation ranges from 1.5–2.2 Gy/fraction; however, SRT delivering substantially higher doses in a small (e.g., ≤5) number of fractions and “hypofractionated” RT typically delivering 40–50 Gy over 5–10 fractions have been increasingly used for aggressive local therapy for select cases of metastatic NSCLC [4, 22, 26]. This shift towards higher RT doses per fraction for select patients with stage IV NSCLC has been supported by mouse models wherein higher RT doses in fewer fractions were associated with increased tumor CD8+ T-cell infiltration [27, 28]. Although these preclinical data are somewhat inconsistent with the results of Shaverdian et al.’s secondary analysis of KEYNOTE-001 showing improved oncologic outcomes for predominantly non-SRT radiation (*n* = 49/70, 70%) followed by pembrolizumab, they offer a potential mechanistic explanation for several observations in our data including (1) reduced OS for EBRT+immunotherapy, (2) an impressive median OS of 32.1 months for high-BED SRT + immunotherapy, and (3) a significant interaction for BED and systemic therapy among patients receiving SRT. Of note, it is possible that the relative influence of dose and fractionation on the biological interactions between RT and immunotherapy may differ as a function of treatment setting since conventionally fractionated EBRT followed by durvalumab has been associated with improved OS for patients with locally advanced rather than metastatic NSCLC [29].

Areas for future investigation include elucidation of RT’s immunomodulatory effects on the tumor microenvironment, determination of clinically optimal radioimmunotherapy combinations/sequencing for metastatic NSCLC, and investigation of the toxicity of combined radioimmunotherapy. To this end, inclusion of molecular correlative studies on future prospective trials of combined radioimmunotherapy for metastatic NSCLC is imperative. A growing number of early-phase protocols [30–36] including diverse radioimmunotherapy strategies will inevitably work towards this goal and lay the foundations for eventual standard-of-care defining phase III trials. Although reports have suggested that combined thoracic radioimmunotherapy is generally safe [37], continued prospective evaluation is recommended to determine the influence of RT dose/sequencing and patient-related factors on the incidence of grade 3+ toxicity with this treatment approach.

Limitations to this study include the lack of information on biomarkers including PD-L1 status, possibility for coding errors in NCDB data, the inability to examine alternative endpoints of interest including toxicity or PFS, heterogeneity of agents coded as immunotherapy in the NCDB, and selection bias regarding treatment received. It is likely that patient selection factors influenced some associations between aggressive local therapy and improved OS as patients with a lower disease burden including oligometastases may have preferentially received SRT and/or higher-BED SRT regimens. Furthermore, the inclusion of patients receiving heterogeneous RT doses within the EBRT subset may limit generalizations for this group. Despite these caveats, our data demonstrate that SRT associates with improved OS for patients with stage IV NSCLC in the NCDB regardless of systemic treatment.

## Conclusions

Overall, the hypothesis-generating observation of superior OS for patients receiving high-BED SRT and immunotherapy, the reduction in OS for patients receiving EBRT and immunotherapy, and the significant interaction between BED and systemic therapy suggest that RT technique may influence the efficacy of immunotherapy in this setting. These findings strongly support efforts to evaluate optimal multimodality radiation and immunotherapy strategies in prospective randomized trials.

## Abbreviations

BED: biologically effective dose
CI: Confidence interval
CoC: Commission on Cancer
EBRT: External beam radiotherapy
Gy: Gray
HR: Hazard ratio
IQR: Interquartile range
NCDB: National Cancer Database
NSCLC: Non-small-cell lung cancer
OS: Overall survival
PFS: Progression-free survival
RT: Radiotherapy
SRT: Stereotactic radiotherapy
